# Frontal theta/beta ratio changes during TOVA in Egyptian ADHD children

**DOI:** 10.17712/nsj.2017.4.20170067

**Published:** 2017-10

**Authors:** Islam F. Halawa, Basma B. El Sayed, Omnia R. Amin, Nagwa A. Meguid, Ann A. Abdel Kader

**Affiliations:** *From the Department of Human Genetics (Halawa, Meguid), National Research Center, from the Unit of Clinical Neurophysiology (El Sayed, Kader), and from the Department of Psychiatry (Amin), Cairo University, Cairo, Egypt*

## Abstract

**Objective:**

To spot the frontal theta/beta ratio alterations during Tests of Variance of Attention (TOVA) in Egyptian attention deficit hyperactivity disorder (ADHD) children.

**Methods:**

This is a cross sectional study performed in Clinical Neurophysiology Unit, Cairo University, Egypt. It included 2 groups, each of 52 children (one of them with ADHD and the other were normal control). EEG was recorded for every subject during normal relaxing circumstance with eyes opened as well as during TOVA.

**Results:**

Comparing both groups revealed statistically significant difference in the theta/beta ratio in both state (normal relaxing with eyes opened and during TOVA), also we found that the theta/beta ratio decreased in normal group (during concentration) while in the ADHD group it increased with a specific pattern.

**Conclusion:**

The theta/beta ratio can be of value in helping for differential diagnosis in patients presenting with mild ADHD.

Attention deficit hyperactivity disorder (ADHD) is the most commonly identified neurobehavioral complaint in childhood, its frequency is supposed to be 6-7%.^1^ The rhythm of electroencephalography (EEG) waveform, echoes the extent of stimulation of the brain zone underneath the electrode. Slow waveform activity, indicates reduction in blood flow and energy (glucose) consumption in this brain zone. These types of brain electrical activity as well echo the rank of arousal of the individual.^2^ Quantitative EEG (QEEG) bids numerous benefits, it possesses supreme chronological resolution (in millisecond time domain) precise to neuronal evidence handling, embodies non-invasive reflections of excitatory and inhibitory cortical neuronal activity concomitant with ancillary hemodynamic events. Moreover, it is economical and transportable. The spatial resolution has improved considerably as channel extent improved from 20-256.^3^ Many researches focused on describing the neural correlates of ADHD, chiefly signals in QEEG. The majority of these researches synopsize that ADHD exhibit a subordinate power in alpha and beta and a superior power in delta and theta bands, moreover, raised theta/beta ratio was perceived when compared to healthy control.^4^ The aim of this study is to detect the QEEG alterations in the frontal zone throughout Tests of Variance of Attention (TOVA) in children with ADHD compared to healthy control, and to identify signals that can assist in development a credible objective diagnostic test for ADHD.

## Methods

The study took place between November 2013 and April 2014 in the Clinical Neurophysiology Unit, Cairo University, Egypt, where 52 children diagnosed with ADHD were prospectively enrolled. Patients were referred from Child Psychiatry Center in social and preventive medicine (Abu El Reesh hospital), Cairo University, Egypt and special needs clinic in National Research Centre, Cairo, Egypt. The methods were approved by the National research center Medical research Ethics Committee.

Inclusion criteria were as follows 1) Age 8-12 years; (2) male; (3) clinical symptoms and signs of ADHD (attentional and combined subtypes only) according to diagnostic and statistical manual 4th edition- text revised (DSM-IV-TR) criteria.^5^

Exclusion criteria were as follows 1) Children with ADHD on medication; (2) Children with hyperactive only subtype of ADHD; (3) Children with any neurologic, cardiac or any other state that may delay mental development.

Fifty-two age and gender matched normal subjects were included as a control group. Those were enrolled from Gastroenterology Clinic, Abu El Reesh hospital, Cairo University, Egypt. All included individuals were subjected to detailed history taking and full general examination to exclude any medical state that might interfere with the process of the study. The EEG was recorded using a Compudmedics E-series (Sydney, Australia) a PC-controlled 32-channel system. 21 scalp EEG electrodes were applied according to the International 10/20 system using Silver chloride electrodes. Video EEG was continuously monitored by a technologist utilizing a Compudmedics^®^ video-EEG system with Profusion EEG 4^®^ software. The high frequency filter was 70 Hz, time constant 0.3 and screen speed 30 mm/sec. The input signal referenced to the ears was filtered between 0.5-50 Hz, and digitized with 250 Hz sampling rate. Impedance was kept below 5 kOhm for all electrodes. The EEG was recorded for 10 minutes during relaxing state with eyes open. Subjects full relaxation was ensured by performing the recording in recombinant position, on a comfortable bed in a semi dark quite room. Furthermore, EEG was recorded for another 10 minutes while the patient was performing TOVA; in a sitting position, on a comfortable chair, at a fixed distance in front of a computer screen where the targets were presented.

**Figure 1:**
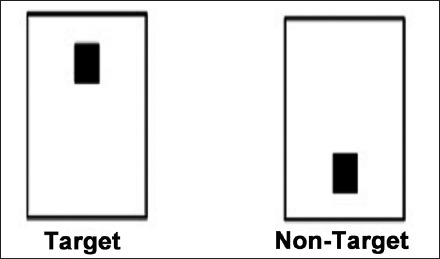
Square on the top (GO trials) left, square on the bottom (NO GO trials) right.

The EEG was monitored by visual inspection for: artifacts (epochs containing artifacts were excluded from the record), presence of any abnormal activity (e.g. sharp waves, slow waves or spike-slow wave complexes) and symmetry between both hemispheres. Then Quantitative data of the frontal zone (right, left and mid frontal areas) were analyzed using WinEEG software. Before processing, epochs of the filtered EEG with large amplitude (>100µV) and/or very fast (>35µV in 20 to 35Hz band) and slow (>50µV in 0 to 1 Hz band) frequency activities were marked and removed from further analysis. The band ranges for theta and beta were set at 4-8 Hz (theta) and 13–21 Hz (beta) registered at Cz, in the eyes-opened (EO) state. Absolute power of these measures were used in the statistical analysis.^6^ The GO/NO GO task (Test of variants of attention, TOVA) is a simple concept, where, trials entailed presenting square on the top (GO trials) or square on the bottom (NO GO trials) (**Figure 1**). The assignment is to push a button as fast as possible in response to all GO trials. During the assignment, patients are seated 1.5 m in front of a computer monitor. The stimuli are displayed on a 19-inch screen using Psytask (Mitsar Ltd, Moscow, Russia). In addition to EEG recordings, behavioral data including omission errors, commission errors, and reaction time are also registered.

The study was approved by the research committee at Cairo University. The parents or caregivers of all patients and controls involved in the study signed written informed consent.

### Statistics

Descriptive results were presented as mean and standard deviation. Comparisons of quantitative variables between the studied groups were carried out using Mann Whitney test. Comparisons in between groups were carried out using Bonferroni multiple comparisons test. Correlation between variables was carried out using Pearson’s correlation. A *p*-value<0.05 was considered statistically significant. All statistical calculations were carried out using the IBM SPSS Statistics for Windows version 22.0 (IBMCorp, Armonk, NY, USA).

## Results

The current study included 104 male subjects, 52 ADHD patients and 52 normal control, their ages ranged between 8-12 years. Where, the mean age of patients group was 9.5±1.11 and that of control group was 9.8±1.16. There was no statistically significant difference between them.

**Figure 2:**
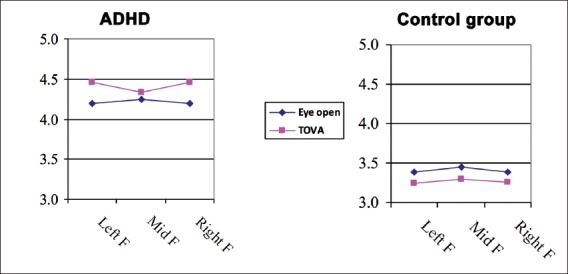
Mean Theta/beta ratio in ADHD group (left) and control group (right). Left F-left frontal area, Mid F-mid frontal area, Right F-right frontal area

The patients group showed significantly higher theta/beta ratio during both eyes open and TOVA states compared to the control group (**Table 1** and **Figure 2**). In TOVA, the ADHD group showed statistically significant higher values compared to the control group, where the mean values for omission were: 10.7±2.74 and 6.25±2.69 with *p*<0.0001, for commission were 35.75±4.64 and 24.03±4.98 with *p*<0.0001, while they were 431.5±22.37 and 385.9±20.9 respectively with *p*<0.0001 for reaction time.

**Table 1:** Mean Theta/beta ratio in frontal zones during eye open and C TOVA in ADHD patients and control groups.

Groups	Eye open*	TOVA* Mean±SD
***Mid frontal zone***		
ADHD	4.25±0.57	4.33±0.61
Control	3.45±0.33	3.3±0.31
***Right frontal zone***		
ADHD	4.2±0.57	4.45±0.61
Control	3.39±0.32	3.26±0.29
***Left frontal zone***		
ADHD	4.19±0.56	4.46±0.62
Control	3.38±0.32	3.24±0.29

TOVA - tests of variance of attention, ADHD - Attention deficit hyperactivity disorder, p-value is <0.0001

Theta/beta ratio ; mid frontal, right as well as left frontal brain areas; in ADHD group during TOVA showed significant increase compared to control group during eye open relaxed state as well as during TOVA, (**Table 2**).

**Table 2:** Multiple comparisons between studied groups (during eye open relaxed state and during performance of TOVA).

Theta/beta ratio	Compared groups	*P*-value
***Mid Frontal***		
ADHD (eye open)	Control (eye open)	0.000
	ADHD (TOVA)	0.365
	Control (TOVA)	0.000
Control (eye open)	ADHD (TOVA)	0.000
	Control (TOVA)	0.128
ADHD (TOVA)	Control (TOVA)	0.000
***Right Frontal***		
ADHD (eye open)	Control (eye open)	0.000
	ADHD (TOVA)	0.006
	Control (TOVA)	0.000
Control(eye open)	ADHD (TOVA)	0.000
	Control (TOVA)	0.164
ADHD (TOVA)	Control (TOVA)	0.000
***Left Frontal***		
ADHD(eye open)	Control (eye open)	0.000
	ADHD (TOVA)	0.005
	Control (TOVA)	0.000
Control(eye open)	ADHD (TOVA)	0.000
	Control (TOVA)	0.120
ADHD (TOVA)	Control (TOVA)	0.000

TOVA - tests of variance of attention, ADHD - attention deficit hyperactivity disorder

In patients group, there was a significant negative correlation between age and mean theta/beta ratio among right where r=-0.681 (*p*<0.0001), left r=-0.69 (*p*<0.0001) and mid frontal zones r=-0.711 (*p*<0.0001) in both eye open relaxing state and during TOVA among right where r=-0.707 (*p*<0.0001), left r=-0.726 (*p*<0.0001) and mid frontal zones r=-0.739 (*p*<0.0001). The same correlation trend was also detected between age and omission error in TOVA, where r=-0.579 (*p*<0.0001). There was a significant positive correlation between omission error and mean theta/beta ratio among right where r=0.331 (*p*=0.024), left r=0.338 (*p*=0.014) and mid frontal zones r=0.340 (*p*=0.014) in both eye open relaxing state, as well as during TOVA among right where r=0.4 (*p*=0.003), left r=0.43 (*p*=0.001) and mid frontal zones r=0.396 (*p*=0.004) .

## Discussion

Current research findings imply that the majority of children with ADHD display quite unswerving EEG alterations in brain electrical activity when compared with normal children, chiefly with respect to their abundant mid frontal theta (4-7 Hz) activity during relaxing state, a divergence demonstrating lessened cortical activity that may possibly be tracked to under arousal.^7^,^8^

A current meta-analysis of 9 research papers with a combined sample of 1,498 ADHD children found an average excess of 32% in theta band power relative to controls.^6^ Nevertheless, eminent theta power alone may be a nonspecific indicator of cortical ailment as indefinite pathology shared with several other conditions including substance abuse, epileptic and bipolar disorders. However, the theta/beta ratio is regarded as unswerving benchmark for discriminating ADHD from control children.^9^ The aim of this study was to identify altered theta/beta ratio during relaxing state and attention (during TOVA) to enhance the capability of the QEEG in detecting ADHD. Since our sample was age and gender matched from the beginning, there were no age and gender significant differences. This denotes that the studied samples were uniform. We selected only males in the studied groups, in agreement with Voeller^10^ who reported that the predominance of ADHD amid boys to girls was 6:1.

During relaxing state, we detected an increase in theta power in the patients compared with control group. This may possibly point to hindered maturation. As reduced speed of the background activity throughout wakefulness is customary till age of 3 years, afterwards alpha waves ought to dominate, and this perseverance of theta waves beyond age of 8 is counted odd. This comes in line with maturational-lag model of ADHD, which clarified that ADHD is coupled with tardy maturity of the brain, presenting traits of younger ages. Also, since theta/beta level reflects brain attention and vigilance. This slowing and increase in theta power may point to under-arousal of ADHD children.^11^ The results showed that the TOVA induced differences in the theta/beta ratio with a significant different effect in ADHD group.

During TOVA, the control group showed reduction in mean theta/beta ratio in all studied frontal zones which can be explained by increased attention and thus increased power of faster waves as beta and a decreased power of slow waves as theta power. While, mean theta/beta ratio increased in children with ADHD. This opposite pattern signifies different functional activation response, where, attention or brain stimulation implies development of more slowing in patients group.

Moreover, we observed that, during TOVA, the theta/beta ratio in mid frontal zone was lower than that in right and left frontal zones in patients group, whereas, it remained higher in control group (**Figure 2**). However, in relaxing state there was no difference between patients and control group in this respect i.e. the reversal of the pattern noticed in the ADHD group was only aggravated by the TOVA.

From this topographical differences between states we can deduce that specific central frontal brain structures are affected in ADHD and they are responsible for concentration and mission carrying out during TOVA.Theta and beta activities changed during eyes opened and TOVA states in both groups, comes in line with EEG topographies reported by Barry et al.^11^ Concerning TOVA results, the ADHD group had significant higher omission, commission errors and reaction time than that of control group. This denotes reduced attention and increased restlessness in ADHD patients.

Among patients group, there was a significant negative correlation between age on one side and mean theta/beta ratio among frontal zones during both states and omission error, on the other side. Which, can be explained by maturation of the brain by aging causing amelioration of ADHD signs, explaining the disappearance of ADHD symptoms around teens in the majority of patients. Furthermore the omission errors revealed a significantly positive linear correlation with mean theta/beta ratio. This reveals that the main pathology in ADHD children may possibly be tracked down to under-arousal and reduced level of brain attention causing cognitive function disarray. This result is in harmony with outcomes of other studies which observed a significant correlation between the left forehead theta power and the results of TOVA and that reported significantly higher power of theta and response delay error in ADHD patients compared to the control group.^12^

### Study limitations

Relative small sample size and sometimes lack of cooperation from the subjects lead to repetition and even cancellation in some cases. Analysis of only the frontal areas however the data for the rest of the brain areas is available for further analysis and might reflect other topographic patterns in the future.

We conclude that mid frontal neuronal structures responsible for attention are the most affected brain area in ADHD. In addition, mean theta/beta ratio represent a credible objective diagnostic test that can be valuable in differential diagnosis of mild ADHD.
